# Regulatory T cells promote functional recovery after spinal cord injury by alleviating microglia inflammation via STAT3 inhibition

**DOI:** 10.1111/cns.14161

**Published:** 2023-03-13

**Authors:** Rui Liu, Ying Li, Ziyue Wang, Peng Chen, Yi Xie, Wensheng Qu, Minghuan Wang, Zhiyuan Yu, Xiang Luo

**Affiliations:** ^1^ Department of Neurology, Tongji Medical College, Tongji Hospital Huazhong University of Science and Technology Wuhan China; ^2^ Hubei Key Laboratory of Neural Injury and Functional Reconstruction Huazhong University of Science and Technology Wuhan China

**Keywords:** injured microenvironment, microglia, neuroinflammation, spinal cord injury, STAT3, Treg

## Abstract

**Background:**

Immediately after spinal trauma, immune cells, and proinflammatory cytokines infiltrate the spinal cord and disrupt the focal microenvironment, which impedes axon regeneration and functional recovery. Previous studies have reported that regulatory T cells (Tregs) enter the central nervous system and exert immunosuppressive effects on microglia during multiple sclerosis and stroke. However, whether and how Tregs interact with microglia and modulate injured microenvironments after spinal cord injury (SCI) remains unknown.

**Method:**

Regulatory T cells spatiotemporal characteristics were analyzed in a mouse contusion SCI model. Microglia activation status was evaluated by immunostaining and RNA sequencing. Cytokine production in injured spinal cord was examined using Luminex. The role of STAT3 in Treg–microglia crosstalk was investigated in a transwell system with isolated Tregs and primary microglia.

**Results:**

Regulatory T cells infiltration of the spinal cord peaked on day 7 after SCI. Treg depletion promoted microglia switch to a proinflammatory phenotype. Inflammation‐related genes, such as *ApoD*, as well as downstream cytokines IL‐6 and TNF‐α were upregulated in microglia in Treg‐depleted mice. STAT3 inhibition was involved in Treg–microglia crosstalk, and STAT3 chemical blockade improved function recovery in Treg‐depleted mice.

**Conclusion:**

Our results suggest that Tregs promote functional recovery after SCI by alleviating microglia inflammatory reaction via STAT3.

## Graphical abstract

Regulatory T cells suppress STAT3 pathway activation and downregulate TNF‐α and IL‐6 expression in microglia, eventually improve the injured spinal microenvironment and promote neural function recovery after spinal cord injury.

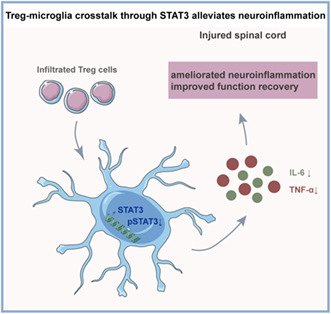

## 1 INTRODUCTION

Spinal cord injury (SCI) often leads to permanent paraplegia; therefore, it represents a major global health priority.^1^, ^2^ Immediately following a spinal trauma, the breakdown of the blood–spinal cord barrier causes an influx of immune cells and proinflammatory cytokines, such as TNF‐α and IL‐1β, into the spinal cord, which triggers an inflammatory cascade.^3^ This inflammation eventually unbalances the injured microenvironment and impedes neural regeneration.^4^ Therefore, preserving a stable microenvironment to limit neuroinflammation and facilitate axonal outgrowth is a promising strategy for SCI treatment.

Previous studies have revealed a dramatic increase in microglial populations at the epicenter of lesions during the first 7 days following SCI.^5^ SCI subacute phase is dominated by M1 microglia, which contribute to cytotoxic damage and demyelination through the generation of reactive oxygen species and proinflammatory cytokines, such as iNOS, TNF‐α, IL‐1β, and IL‐6.^6^ However, during the chronic phase, M2 microglia become prominent and promote regeneration through the production of the anti‐inflammatory cytokine IL‐10.^7^ A recent study has shown that shifting microglia toward an anti‐inflammatory state improves locomotive function and electrophysiological transmission after SCI.^8^ Enhancing microglial dispersion promotes wound compaction and motosensory recovery following SCI.^9^ Moreover, transplantation of proteinase inhibitor–treated microglia into the spinal cord lesions significantly improves wound healing and axon regeneration.^10^ These findings suggest microglia as a therapeutic target to regulate injured spinal microenvironment and promote functional recovery after SCI.^11^

Tregs are a subset of CD4^+^ T cells expressing the transcription factor FOXP3. Upon injuries or diseases, Tregs enter the central nervous system (CNS) and preserve the immune homeostasis.^12^ Emerging evidence suggests that Tregs could directly modulate microglia's activity and protect against pathological neuroinflammation.^13^ The expansion of endogenous Tregs in a mouse model of amyotrophic lateral sclerosis (ALS) could reset the homeostatic functions of the microglia, thereby prolonging survival time.^14^, ^15^ Further, in mice with Treg‐specific gene knockouts or Treg dysfunctions, the brain microglia appeared more activated, with higher expression of GM‐CSF and TNF‐α during experimental autoimmune encephalomyelitis (EAE).^16^, ^17^, ^18^ Tregs in ischemic brain polarize microglia toward reparative phenotypes that promote white matter repair during both acute and chronic stages.^19^, ^20^, ^21^ The immunoregulatory effects of Tregs on microglia were further supported by in vitro studies where Tregs, through secretion of anti‐inflammatory cytokines (IL‐10, IL‐35, TGF‐β), were shown to directly suppress the production of proinflammatory cytokines and chemokines by cultured microglia.^22^, ^23^ Previous studies have reported that treatment with IL‐10, VX‐765, or CCL28 increases Treg numbers and reinforces functional recovery after SCI.^24^, ^25^, ^26^ However, whether and how Tregs interact with microglia and modulate injured microenvironment after SCI remains unknown. Recent studies have revealed that Tregs suppress the IL‐6–STAT3 signaling pathway in a mouse model of middle cerebral artery occlusion.^27^ STAT3 activation promotes disease‐associated microglia activation during late‐onset Alzheimer's disease, and is increased after SCI.^28^, ^29^, ^30^ Yet, only few studies tracked the association between the STAT3 pathway and Tregs' beneficial effect on microglia after SCI.

In this study, we analyzed Treg population dynamics during mouse contusion SCI. Tregs were selectively depleted to formally test whether and how Tregs regulate microglia activation and limit neuroinflammation following SCI. Our results showed that Treg depletion resulted in microglial activation and a phenotypic switch via STAT3 pathway, which eventually enhanced the proinflammatory microenvironment caused by SCI. STAT3 inhibition suppressed microglia‐mediated neuroinflammation and promoted neural function recovery after SCI in Treg‐depleted mice. Our findings provide insights into the pathway underlying neuronal damages after SCI and reveal STAT3 as a possible target to explain Tregs' beneficial effect on recovery.

## 2 MATERIALS AND METHODS

### 2.1 Animals

Wild‐type C57BL/6J mice, *Foxp3‐EGFP* transgenic mice, and *Foxp3‐DTR* transgenic mice were purchased from Shanghai Model Organisms Center. *Foxp3‐EGFP* mice co‐express EGFP and the regulatory T cell‐specific transcription factor *Foxp3*, which could accurately identify the FOXP3^+^ T cell population.^31^ Depletion of Tregs was performed in Foxp3‐DTR mice by intraperitoneal injection of Diphtheria toxin (DT, 40 μg/kg body weight) 3 days prior to SCI, and was repeated every 3 days to maintain Treg cell depletion until sacrifice.^32^ Female mice (12‐week‐old, weighing 22–25 g) were used for the experiments in vivo, given that male rodents are not often used when modeling SCI due to more‐severe postoperative complications, such as urinary and bowel incontinence and urinary tract infections^33^; these male‐specific complications increase mortality and adverse health issues.^34^ Analysis of NIH‐funded, rodent, primary research publications demonstrated that females were the sole sex used in the majority of SCI experiments.^35^ All animal procedures were approved by the Ethics Committee of the Institutional Animal Care and Use Committee of Tongji Medical college, Huazhong University Science and Technology University (approval number: TJH‐202201003).

### 2.2 SCI model

Prior to surgery, the mice were anesthetized with isoflurane. A T9‐11 laminectomy was performed, and the spinal cord was exposed at T10. A 5‐g weight was dropped from a height of 11 mm onto the exposed dorsal surface of the spinal cord using a modified NYC impactor (J$K Seiko Electronic). Sham‐treated mice underwent a laminectomy at T9‐11 without SCI. Basso mouse scale (BMS) was used to evaluate the recovery of motor function in mice at 1, 3, 7, 14, 21, and 28 days after injury. Detailed information about BMS testing is further described in Appendix S1.

### 2.3 Flow cytometry

Briefly, the single‐cell suspensions were first incubated with Fc blocker (anti‐CD16/32 antibody) for 15 min. For surface antibodies, such as CD4 and CD25, isolated cells were incubated for 30 min at 4°C in the dark (1:200; BD Biosciences). For intranuclear staining of *Foxp3*, *Foxp3‐*EGFP mice were used. The samples were measured on a BD FACSAriaTM flow cytometer (BD Biosciences) and the data were analyzed in FlowJo software.

### 2.4 Immunofluorescence

For immunofluorescence staining, the following primary antibodies were used: rabbit anti‐calcium binding adaptor molecule 1 (IBA1) (Wako), goat anti‐GFAP and anti‐NeuN (Abcam), mouse anti‐IL‐6, rabbit anti‐TNF‐α (Santa Cruz), rat anti‐KI67 (Abcam), mouse anti‐FOXP3 (BD), mouse anti‐CD16, and anti‐CD206 (Boster). The slices were imaged using a confocal microscope (Olympus FV1000). Three‐dimensional reconstruction and co‐localization analysis were conducted using Imaris software. Morphological changes in microglia were analyzed with the Sholl analysis plugin for ImageJ. Detailed information about three‐dimensional reconstruction and measurement of the co‐localization coefficient using Imaris software, and the Sholl analysis is thoroughly demonstrated in Appendix S1.

### 2.5 Sequencing of microglia RNA

Microglia were separated from spinal cord as described before.^27^ Briefly, the single‐cell suspensions were incubated with anti‐CD45‐PerCP and anti‐CD11b‐APC antibodies for 30 min at 4°C in the dark (1:200; BD Bioscience). The CD45^int^CD11b^high^ cells were sorted on FACSAriaTM (BD Bioscience). For each sample, 200 cells were collected, and total RNA was extracted using RNeasy Micro kit (QIAGEN, GER). RNA sequencing was performed at Beijing Genomics Institute as described in Yuan et al.^36^ on the BGISEQ500 platform (BGI).

### 2.6 Cytokine assay

CCL2, CCL3, GM‐CSF, MIP‐1 alpha, IFN‐gamma, TIM‐1, IL‐1 beta, IL‐3, TNF‐alpha, VEGF, IL‐5, IL‐10, IL‐13, IL‐33, MMP‐2, MMP‐9, IL‐17, IL‐12 p70, IL‐6, TNF RII, IL‐4, IL‐2, IL‐1 alpha, and ICAM‐1 levels in injured tissues were measured using Mouse Magnetic Luminex® Assays (R&D Systems). Enzyme‐linked immunosorbent assays (ELISA) kits were used to quantify IL‐6 and TNF‐α levels (Neobioscience) in cell culture supernatants.

### 2.7 RNA extraction and qPCR

Total RNA from tissues or primary microglia was extracted with TRIzol (Invitrogen). cDNA was synthetized using a ReverTra Ace qPCR RT Kit (Toyobo). Quantitative real‐time PCR (qRT‐PCR) was performed using BioRad CFX Connect and SYBR Green PCR Master Mix (Toyobo). Expression data were normalized to the internal control GAPDH. The primer sequences are listed in Table S1.

### 2.8 Western blotting

Proteins extracted from spinal cord tissues and primary microglia were separated by SDS‐PAGE and transferred onto nitrocellulose filters membranes. The membranes were blocked for 1 h at room temperature using 5% nonfat milk, and incubated overnight at 4°C with primary antibodies (1:1000). The following primary antibodies were used: rabbit anti‐STAT3 and anti‐phospho‐STAT3 (Cell Signaling), mouse anti‐IL‐6 and rabbit anti‐TNF‐α (Santa Cruz), rabbit anti‐β‐ACTIN and rabbit anti‐GAPDH (Servicebio). Next, the membranes were incubated with secondary antibodies for 1 h at room temperature (1:1000; Servicebio). Protein expression levels were normalized to β‐ACTIN or GAPDH internal controls.

### 2.9 Primary microglia culture

Briefly, brains from postnatal 1‐ to 3‐day‐old mice were diced after removing the meninges. The dissociated cells were plated onto PDL‐coated T‐flasks filled with culture medium (DMEM/F12 containing 20% heat‐inactivated fetal bovine serum [FBS], 1% penicillin, and 1% streptomycin). The cells were grown to confluence for 12 days, and microglia were collected by shaking the mixed glia‐containing flasks for 1 h at 180 rpm at 37°C.

### 2.10 In vitro Treg culture and coculture with microglia

Seven days after SCI, mouse spleens were harvested, and CD4^+^CD25^+^ Tregs were isolated using a mouse Treg cell isolation kit (Miltenyi). Isolated Tregs were stimulated with soluble anti‐CD3 (4 μg/mL), anti‐CD28 (5 μg/mL), and IL‐2 (100 ng/mL) for 3 days in Treg culture media (RPMI1640 containing 10% FBS, 1% penicillin, and streptomycin). For Treg cell‐microglia coculture, Tregs (5 × 10^5^ per well) were placed in a transwell insert (Corning, 24 mm) and incubated with primary microglia (2 × 10^6^ per well) located in the lower chamber in microglia culture media for 2 days before treatment.

### 2.11 Oxygen–glucose deprivation/reoxygenation

Oxygen–glucose deprivation/reoxygenation (OGD/R) was used to mimic ischemic injury in vitro, as described previously.^6^ Briefly, the culture medium of primary microglia cultures was replaced with glucose‐free DMEM (Gibco). The cells were placed in a hypoxic incubator (Thermo Scientific) at 94% N_2_, 5% CO_2_, and 1% O_2_ at 37°C for 3 h, and then returned to standard culture medium and incubation conditions for another 3 h (37°C, 95% air, and 5% CO_2_).

### 2.12 Drug treatment

STAT3 inhibitor (SH‐4‐54) was purchased from MedChemExpress. For in vivo treatment, SH‐4‐54 was solubilized in saline containing 10% DMSO, 40% PEG300, and 5% Tween‐80. The mice were injected intraperitoneally with SH‐4‐54 (25 mg/kg) every 3 days for a month before SCI, and the treatment was continued thereafter until sacrifice of the animals. For in vitro study, SH‐4‐54 was dissolved in sterile DMSO. Primary microglia were treated with SH‐4‐54 at concentration of 1 μM 3 days prior to OGD/R.

### 2.13 Luxol fast blue staining

Briefly, spinal cord tissue sections were immersed in 95% ethanol for 5 min at room temperature, followed by overnight incubation in 0.1% LFB (Servicebio) at 60°C. Observation and image acquisition were performed using a light microscope (BX51; Olympus).

### 2.14 Statistical analysis

Graphs and statistical analyses were made using Prism 8 software (GraphPad). For in vivo study, experiments were performed in biological quintuplicate. For in vitro study, experiments were performed in triplicate. The data are shown as mean ± SEM. Kolmogorov–Smirnov test was used to assess data distribution. For normally distributed data, Student's *t* test was used to assess differences between two groups, and one‐way ANOVA was used to analyze differences between three or four datasets. Data that did not exhibit a normal distribution were analyzed using Mann–Whitney *U* test between two groups or Kruskal–Wallis tests between three datasets. *p* values <0.05 were considered statistically significant.

## 3 RESULTS

### 3.1 Tregs infiltrated the injury core area and mainly clustered around microglia after SCI

**FIGURE 1:**
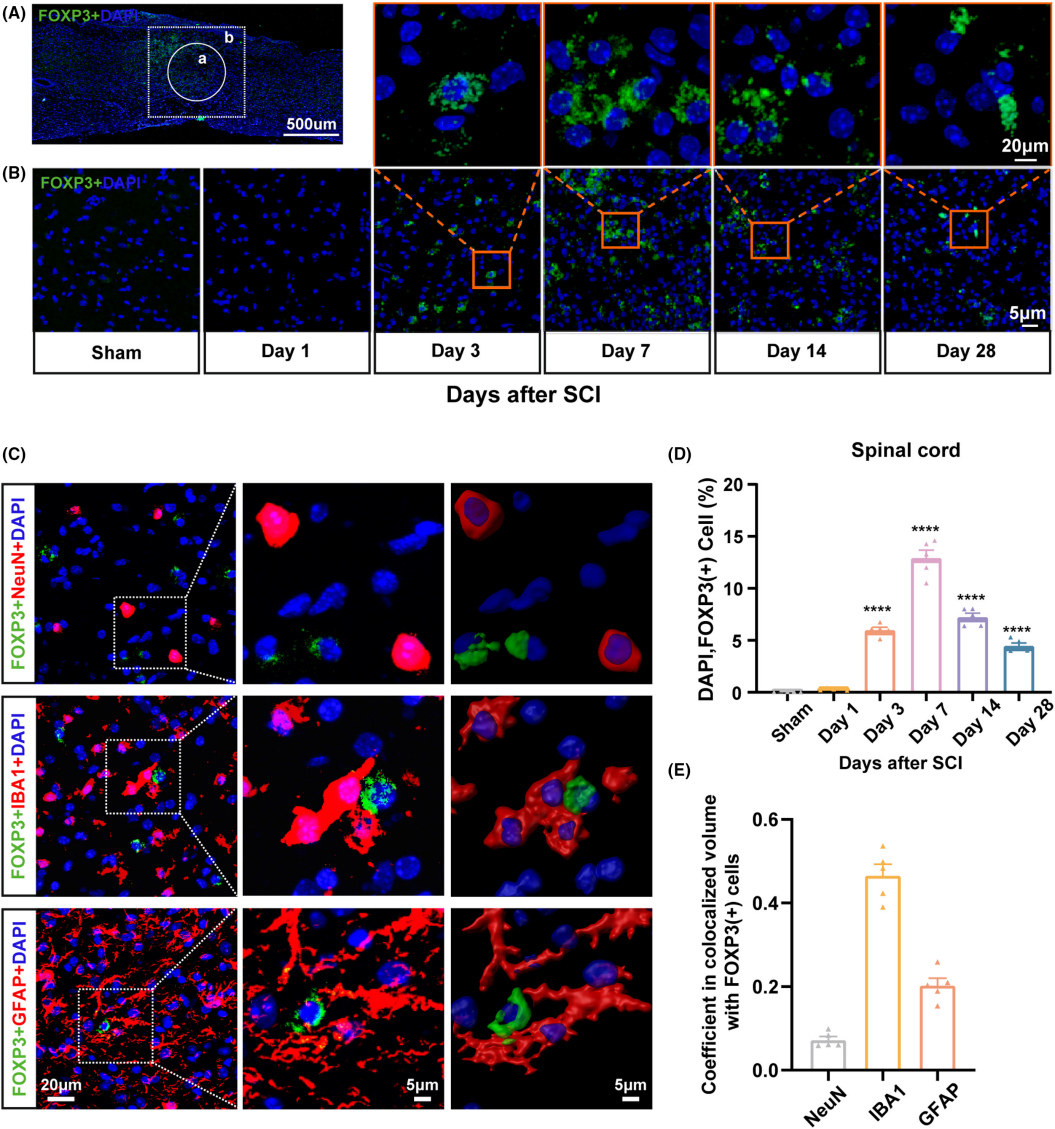
Tregs infiltrated the injury core area and mainly clustered around microglia after spinal cord injury (SCI). (A) Representative images showing the injury core (a) and imaging area (b) for FOXP3 (green) imaging. The location of the injury core after SCI is indicated by white circles (a), while the imaging sites (the area surrounding the injury core) are marked by white frames (b). Scale bar: 500 μm. (B) Representative immunofluorescence images of FOXP3 (green, Tregs) staining of *Foxp3‐EGFP* mouse spinal cord section surrounding the lesion core at different time points following SCI. Scale bars: 20 μm (upper columns); 5 μm (bottom column). (C) Representative images showing the spatial relationship between FOXP3^+^ (Tregs) and NeuN^+^ (neurons) cells (top row), FOXP3^+^ (Tregs) and IBA1^+^ (microglia) cells (middle row), and FOXP3^+^ (Tregs) and GFAP^+^ (astrocytes) cells (bottom row). The three‐dimensional reconstruction (right column) was performed by Imaris 3D imaging. Scale bars: 20 μm (left column); 5 μm (2 right columns). (D) Time course quantification of the proportion of FOXP3^+^ cells surrounding the injury core at day 3, 7, 14, and 28 post‐SCI. (E) Calculation from images on (B) of the colocalization coefficients between FOXP3^+^ staining, and, respectively, NeuN^+^, IBA1^+^, and GFAP^+^ staining by Imaris 3D imaging. Ten to 15 Tregs per mouse were analyzed. Data are represented as the mean ± SEM (*n* = 5 for each group; ****p < 0.001).

To assess the spatiotemporal patterns of Treg after SCI, we first analyzed the CD4^+^CD25^+^FOXP3^+^ Treg population in spleen and blood by flow cytometry in *Foxp3*‐EGFP (GFP) mice. The gating strategy is shown in Figure S1A. We observed an overall increase in splenic and blood Treg number from day 3 until at least 28 days after SCI (*p* < 0.01; Figure S1B–D). This increase was clearly corroborated through immunofluorescence analysis of spinal cord tissues, showing that the number of infiltrating Tregs peaked on day seven after SCI and then gradually decreased, but remained elevated until 28 days after SCI (*p* < 0.001; Figure 1B,D). The locations of the injury core and imaging area for immunofluorescence analysis are presented in Figure 1A. This kinetics suggested a potential role of Tregs in function recovery after SCI.

Confocal analysis by Imaris 3D imaging was used to probe the spatial relationship between FOXP3^+^ (Tregs) and NeuN^+^ (neurons), IBA1^+^ (microglia), and GFAP^+^ (astrocyte) cells at 7 days after SCI (Figure 1C). Three‐dimensional reconstruction and measurement of the co‐localization coefficient between NeuN^+^, IBA1^+^, or GFAP^+^ staining with FOXP3^+^ staining revealed that infiltrated Tregs mainly clustered around microglia and astrocytes (Treg/neuron coefficient: 0.0699; Treg/astrocyte coefficient: 0.2117; Treg/microglia coefficient: 0.4483; Figure 1E). These findings suggest that following SCI, Tregs infiltrate the spinal lesion and interact with CNS resident cells, including microglia and astrocytes.

### 3.2 Treg depletion promoted microglial morphological alterations and phenotype switch after SCI

To further determine the role of Tregs in neural function recovery after SCI, we selectively depleted Tregs in *Foxp3‐DTR* (DTR) transgenic mice. Since the aforementioned experiments indicated that the number of infiltrating Tregs peaked on day seven after SCI, this time point was chosen to study Tregs' impact after SCI. Flow cytometric analysis and immunofluorescence results indicated that Treg numbers were drastically reduced in spleen, blood (Figure S2A–C), and spinal cord (Figure S2D) in DTR + DT mice (*p* < 0.01). IBA1^+^ and GFAP^+^ staining results indicated that microglial staining intensity was further increased in Treg‐depleted mice following SCI (*p* < 0.001), whereas astrocyte staining was equivalent (Figure S2E–G). These results indicated that Treg depletion resulted in increased microglial activation after SCI.

**FIGURE 2:**
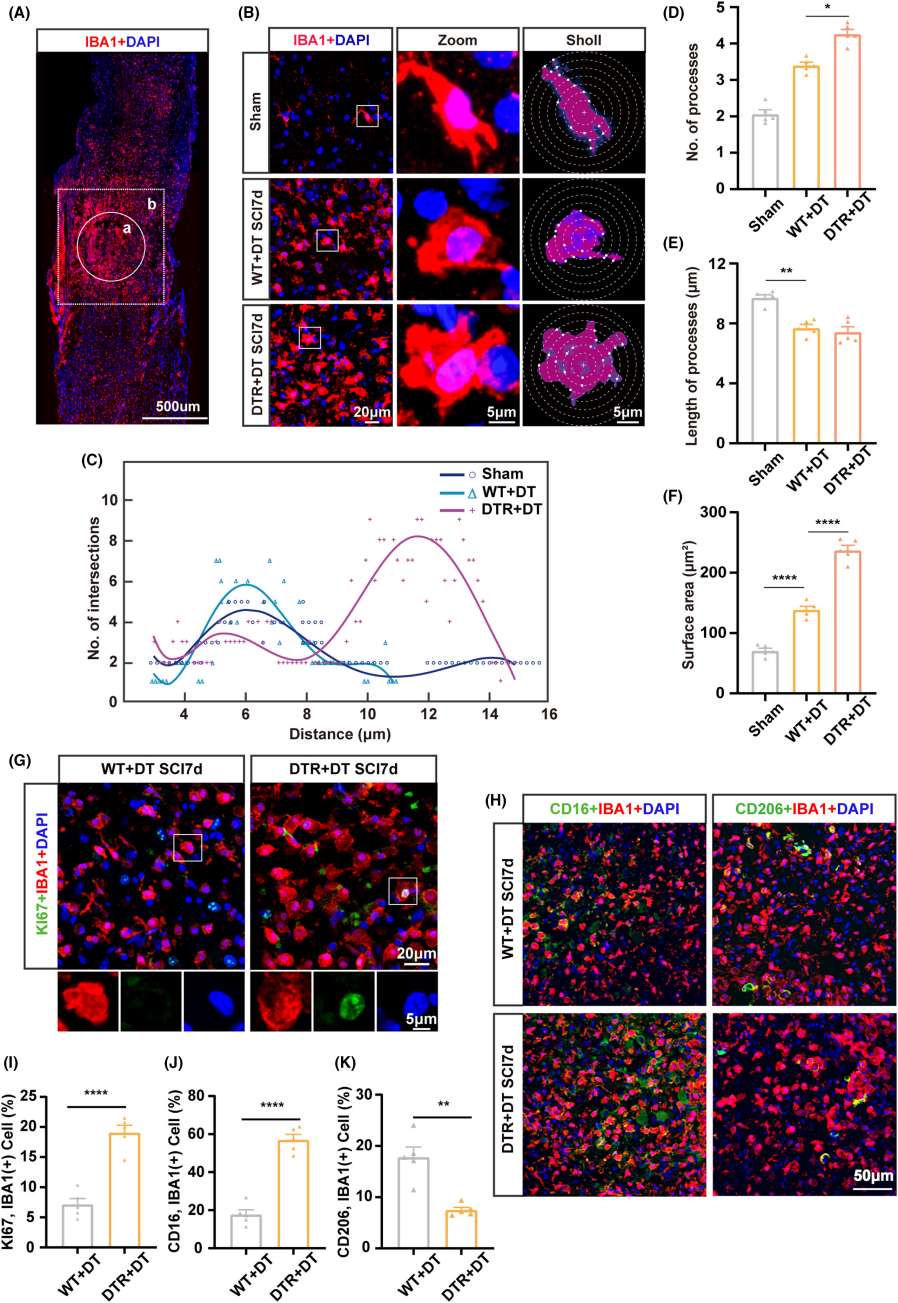
Treg depletion promoted microglial morphological alterations and phenotype switch after spinal cord injury (SCI). (A) Representative images showing the injury core (a) and imaging area (b) for IBA1 (red) imaging. The location of the injury core after SCI is indicated by white circles (a), while the imaging sites (the area surrounding the injury core) are marked by white frames (b). Scale bar: 500 μm. (B) Morphological alterations of the microglia in the spinal lesions of WT (WT + DT) and Treg‐depleted *Foxp3‐DTR* (DTR + DT) mice at day 7 post‐SCI, compared with sham‐operated mice, and assessed by Sholl analysis of IBA1^+^ stained (red) sections using ImageJ. Scale bars: left row, 20 μm; middle and right rows: 5 μm. (C) Non‐linear fitting curves of the average number of microglial branch intersections per 2‐μm concentric shells drawn from the cell soma according to the Sholl method. (D–F) Quantification by the Sholl methods of number (D) and length (E) of microglial processes and soma size (F) in the animals described in (A) and (B). (G) Representative immunofluorescence images showing injury cores on mouse spinal cord sections stained for KI67 (green) and IBA1 (red). Scale bars: top row, 20 μm; bottom row, 5 μm. (H) Representative confocal images of co‐immunostaining for IBA1 (red) with the M1 marker CD16 (green, left column), or the M2 marker CD206 (green, right column) of injured spinal cord sections from WT (WT + DT) and Treg‐depleted *Foxp3‐DTR* (DTR + DT) mice at day 7 post‐SCI. Scale bar: 50 μm. (I–K) Quantification of the percentages of KI67^+^ (I), CD16^+^ M1 (J), or CD206^+^ M2 (K) microglia (IBA1^+^) in injured spinal cord section from WT (WT + DT) or Treg‐depleted *Foxp3‐DTR* (DTR + DT) mice at day 7 post‐SCI. Data are represented as the mean ± SEM (*n* = 5 for each experimental group; *p < 0.05, **p < 0.01, ***p < 0.005, ****p < 0.001).

We then assessed potential morphological changes in IBAI^+^ microglia caused by Treg depletion by Sholl analysis. The imaging area for Sholl analysis is shown in Figure 2A. In the presence of Tregs and after SCI, microglia adopted an amoeboid phenotype with enlarged soma and shorter processes compared with uninjured microglia (*p* < 0.05; Figure 2B,D–F). However, the main difference was observed upon Treg depletion, consisting of increased number of processes (*p* < 0.05; Figure 2D) and larger soma size (*p* < 0.001; Figure 2F) compared with WT mice (Figure 2B), as indicated by the non‐linear curve fit of the average number of microglial branch intersections (Figure 2C). In contrast, the absence of Tregs did not seem to influence process length (Figure 2E).

The amoeboid phenotype reflects a shift in microglial function with increased proliferation ability and neurotoxicity.^37^ Therefore, we assessed microglial proliferation by co‐immunostaining of IBA1 and KI67 (Figure 2G). We observed that on day seven after SCI, microglia in Treg‐depleted mice exhibited a higher proliferative ability (*p* < 0.001; Figure 2I). Microglial activity is typically categorized as neurotoxic (M1) or neuroprotective (M2).^38^ To compare the phenotype of microglia in the presence or absence of Tregs, we performed co‐immunostaining of spinal sections for IBA1 with the M1 marker CD16, or for IBA1 with the M2 marker CD206 (Figure 2H). This analysis showed increased numbers of CD16^+^IBA1^+^ M1 cells in Treg‐depleted mice compared with WT mice (*p* < 0.001; Figure 2J), accompanied by a reciprocal decrease in CD206^+^IBA1^+^ M2 cell number (*p* < 0.01; Figure 2K). These results showed that by day 7 post‐SCI, Treg depletion had favored the acquisition of an M1 neurotoxic phenotype over a M2 protective phenotype by microglia.

### 3.3 Treg depletion resulted in enhanced microglia‐mediated proinflammatory microenvironment after SCI

**FIGURE 3:**
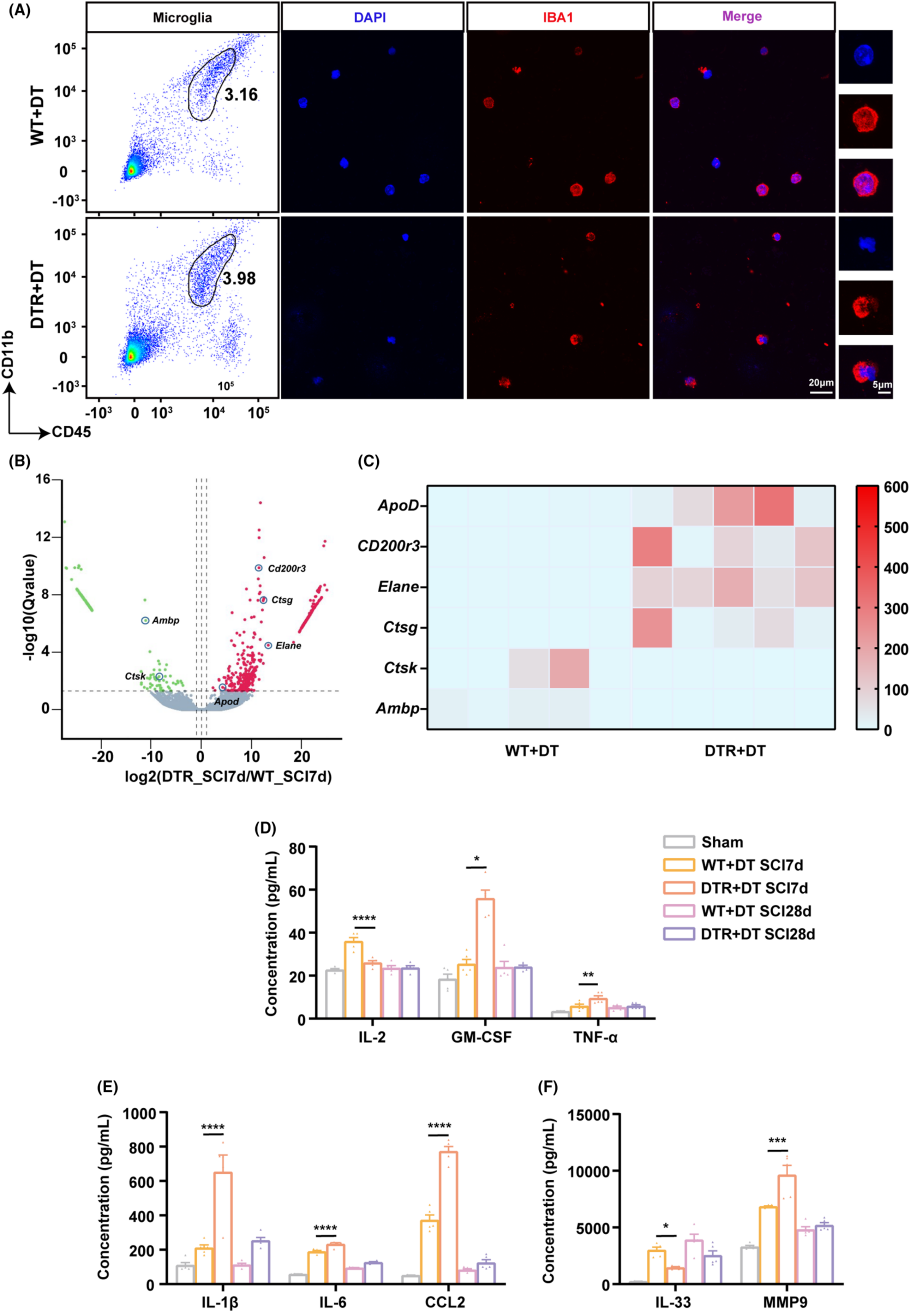
Treg depletion resulted in enhanced microglia‐mediated proinflammatory microenvironment after spinal cord injury (SCI). (A) Representative flow cytometric plots showing the gating strategy of CD45^int^CD11b^high^ microglia isolation from injured spinal cords of WT (WT + DT) and Treg‐depleted *Foxp3‐DTR* (DTR + DT) mice at day 7 post‐SCI. Representative immunofluorescence image of FACS‐sorted microglia stained for IBA1 for purity evaluation. Scale bar: three left columns: 20 μm; right column: 5 μm. (B) Volcano plot comparing gene expression in microglia from Treg‐depleted mice vs. WT mice at day 7 post‐SCI. Differentially expressed genes related to pro‐inflammatory (*ApoD*, *Elane*, *Ctsg*, and *Cd200r3*) or anti‐inflammatory (*Ctsk* and *Ambp*) microglial activities are indicated. (C) Heat map representation showing the expression of the differentially expressed genes highlighted in (B). (D–F) Expression of inflammatory and anti‐inflammatory cytokines related to aforementioned differentiated genes by microglia isolated from sham‐operated, and DT‐treated WT (WT + DT) or Foxp3‐DTR (DTR + DT) mice, assessed by Luminex at day 7 and 28 post‐SCI. Data are represented as the mean ± SEM (*n* = 5 for each group; *p < 0.05, **p < 0.01, ***p < 0.005, ****p < 0.001).

Increasing evidence supports that a dichotomized classification of microglia may not reflect the phenotypic diversity of these cells.^38^ To better understand how Tregs influence microglia's function, microglia were sorted by flow cytometry from injured spinal cords of WT and Treg‐depleted DTR mice 7 days after SCI. The purity of the sorted cells was confirmed by immunofluorescence microscopy (Figure 3A). The sorted cells+ were analyzed by RNA sequencing to determine the transcriptional changes induced in microglia by Treg depletion. The Volcano plot and heatmap analysis revealed a significant upregulation of inflammation‐related genes, including *ApoD*, *Elane*, *Ctsg*, and *Cd200r3* in Treg‐depleted mice (Figure 3B,C). These results indicated that Treg depletion favored the upregulation of inflammatory gene expression in microglia after SCI.

To further investigate whether Treg depletion favors a neuroinflammatory microglial responses and enhances the pro‐inflammatory microenvironment provoked by SCI, we measured the levels of pro‐ and anti‐inflammatory cytokines linked to aforementioned differentially expressed genes using Luminex in spinal cord taken on days 7 and 28 after SCI. GM‐CSF, TNF‐α, IL‐1β, IL‐6, CCL2, and MMP‐9 levels were markedly increased in DTR mice at day 7 compared with WT mice. In contrast, the levels of anti‐inflammatory IL‐2 and IL‐33 were significantly decreased (*p* < 0.001; Figure 3D–F). On day 28 after SCI, there was no difference in the level of these cytokines between the two groups (Figure 3D–F). Thus, Treg depletion resulted in increased expression of inflammatory factor at day 7 post‐SCI, thereby promoting a proinflammatory microenvironment.

### 3.4 Treg depletion increased IL‐6 and TNF‐α expression in microglia after SCI

**FIGURE 4:**
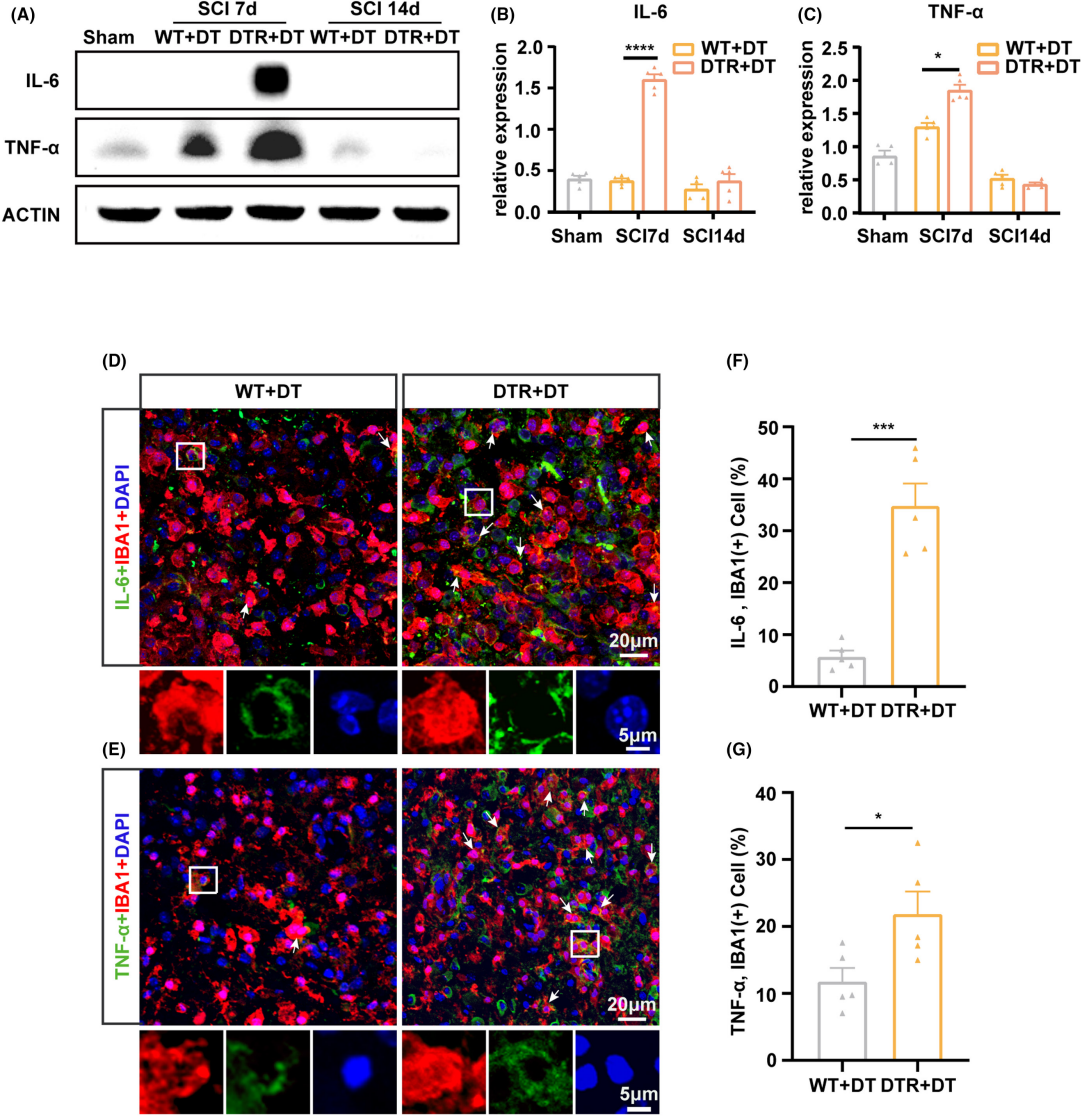
Treg depletion increased IL‐6 and TNF‐α expression in microglia after spinal cord injury (SCI). (A) Western‐blot analysis of IL‐6 and TNF‐α level day 7 after SCI, in DT‐treated WT (WT) and *Foxp3‐DTR* (DTR) mice at days 7 (7d) and 14 (14d) post‐SCI. (B, C) Quantification of IL‐6 (B) and TNF‐α (C) by western blot, normalized to ACTIN. (D, E) Representative images of injured spinal cord sections from DT‐treated WT (WT + DT) or *Foxp3‐DTR* (DTR + DT) mice, 7 days post‐SCI, double‐stained for IL‐6 (D) or TNF‐α (E) (green) and IBA1 (red). IL‐6^+^IBA1^+^ and TNF‐α^+^IBA1^+^ cells are indicated by white arrows. Scale bars: Top row, 20 μm; bottom row, 5 μm. (F, G) Percentage of IL‐6^+^IBA1^+^ (F) and TNF‐α^+^IBA1^+^ (G) double‐stained cells on injured spinal cord sections from DT‐treated WT (WT + DT) or *Foxp3‐DTR* (DTR + DT) mice day 7 post‐SCI. Data are represented as the mean ± SEM (*n* = 5 for each group; *p < 0.05, **p < 0.01, ***p < 0.005, ****p < 0.001).

*ApoD* upregulation in microglia after Treg depletion was of particular interest, for it has been associated with TNF‐α and IL‐6 production and aggravated neuroinflammation.^39^ Therefore, we sought to assess whether Treg depletion enhanced TNF‐α and IL‐6 production by microglia. Quantification by western blot indicated higher levels of IL‐6 (*p* < 0.001; Figure 4B) and TNF‐α (*p* < 0.05; Figure 4C) in DTR mice at day 7 post‐SCI compared with WT mice. However, day 14 after SCI, no significant difference was observed (Figure 4B,C).

Next, we performed double‐labeling of spinal cord sections for TNF‐α or IL‐6 (green) and IBA1 (red) to confirm that these cytokines were derived from microglia. We found that the proportions of both IL‐6^+^IBA1^+^ (*p* < 0.001; Figure 4D,F) and TNF‐α^+^IBA1^+^ (*p* < 0.05; Figure 4E,G) cells were increased in Treg‐depleted mice 7 days following SCI, suggesting that Tregs may directly repress IL‐6 and TNF‐α expression in microglia after SCI.

### 3.5 Tregs attenuated microglia's inflammatory response by reducing the STAT3 pathway activation in vivo and in vitro

**FIGURE 5:**
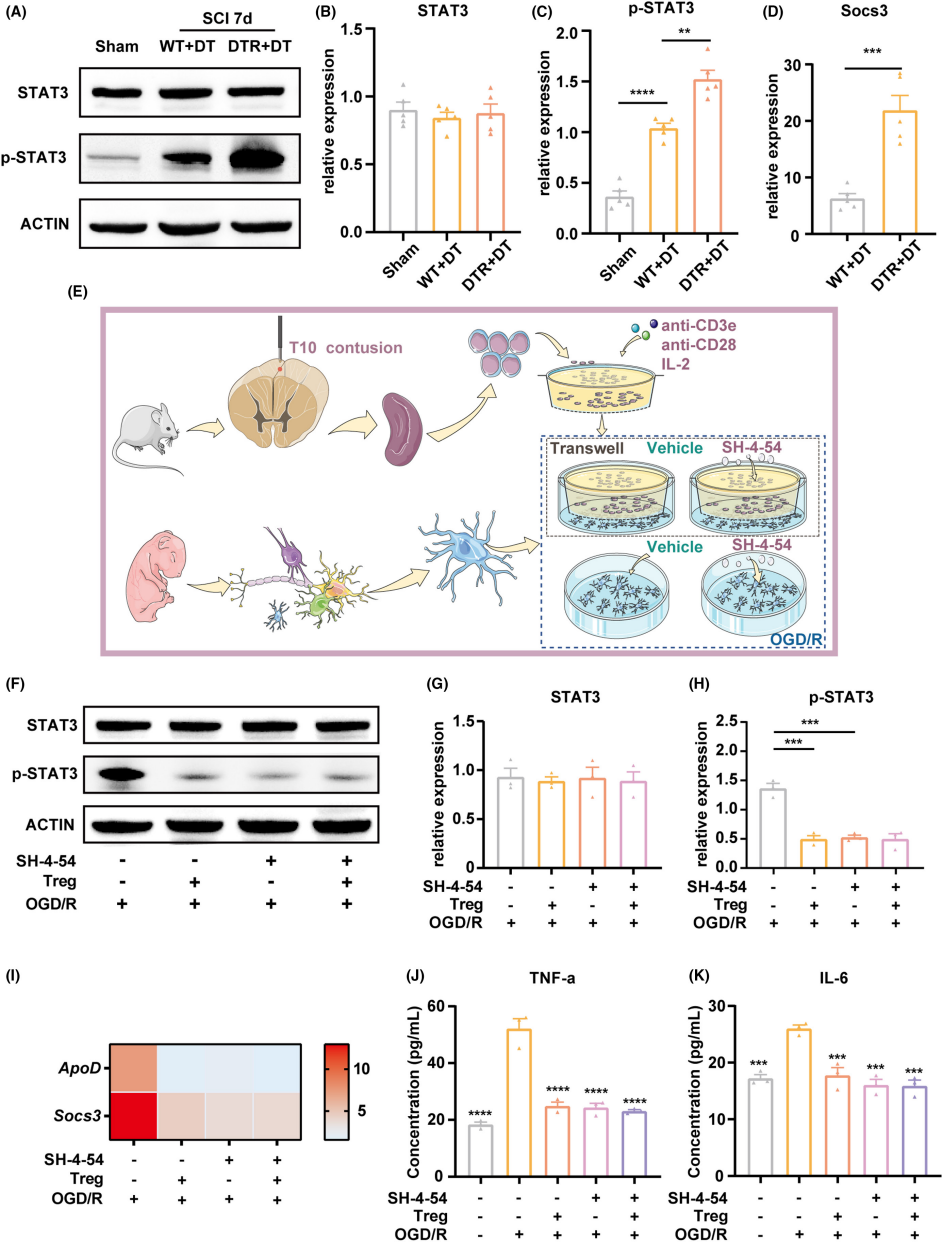
Tregs attenuated microglia's inflammatory response via STAT3 pathway in vivo and in vitro. (A) Western blots measuring total and phosphorylated STAT3 (p‐STAT3, Tyr705) in sham‐operated mice and DT‐treated WT or *Foxp3‐DTR* mice, day 7 post‐SCI. (B, C) Quantification of STAT3 (B) and phosphor‐STAT3 (C) protein by western blot, normalized to ACTIN. (D) Relative expression of *Socs3* mRNA level quantification by qPCR. (E) Design of the Treg cell‐microglia coculture experiment to analyze STAT3 involvement: Tregs were isolated from mouse spleens and stimulated with anti‐CD3e, anti‐CD28, and IL‐2 for 3 days. Tregs were then cocultured for 24 h in transwell inserts with primary microglia placed in the lower compartment, and then submitted to OGD/R conditions. The microglia were treated with SH‐4‐54 or vehicle for 3 days prior to OGD/R. (F) Western blot analysis of STAT3 and p‐STAT3 expression by microglia after different treatments. (G, H) Quantification of STAT3 (G) and p‐STAT3 (H) proteins by western blot normalized to ACTIN. (I) Heatmap showing consistent downregulation of *Apod* and *Socs*3 expression in microglia after Treg‐coculture and/or treatment with SH‐4‐54. (J, K) Quantification of concentration of TNF‐α and IL‐6 in microglia after different treatments measured by ELISA. Data are represented as the mean ± SEM (*n* = 5 for each group; *p < 0.05, **p < 0.01, ***p < 0.005, ****p < 0.001).

Previous research has reported that IL‐6 and TNF‐α activate the transcription factor STAT3 and its downstream targets.^40^ Western blotting showed a significant increase in phosphorylated STAT3 (p‐STAT3) level in Treg‐depleted mice (*p* < 0.01; Figure 5A–C). Consistently, the expression of the STAT3 target gene suppressor of cytokine signaling 3 (*Socs3*) was also increased in Treg‐depleted mice (*p* < 0.001; Figure 5D). These results indicated that in the absence of Tregs, the STAT3 pathway was highly activated following SCI.

To further investigate whether Tregs regulate microglia's activity via repressing the STAT3 pathway, Tregs were cocultured with primary microglia for 24 h, and then submitted to OGD/R. To assess the effect of blocking STAT3 pathway, we made use of SH‐4‐54, a small‐molecular STAT3 inhibitor with high blood–brain barrier permeability.^41^ The microglia were treated with SH‐4‐54 for 3 days prior to OGD/R (Figure 5E). The relative expression of p‐STAT3 protein and *Socs3* mRNA was reduced significantly in microglia cocultured with Tregs (*p* < 0.001; Figure 5F–I). Similarly, and as expected, SH‐4‐54 treatment effectively blocked OGD/R‐induced STAT3 phosphorylation (*p* < 0.001; Figure 5F–I). Tregs or STAT3 inhibition reduced the expression of *ApoD* (*p* < 0.001; Figure 5I), TNF‐α (*p* < 0.001; Figure 5J), and IL‐6 (*p* < 0.001; Figure 5K) upon OGD/R. Combining Treg and SH‐4‐54 treatment did not further downregulate the expression of these molecules in microglia, compared with Tregs or SH‐4‐54 used alone (*p* < 0.001; Figure 5F–K). Collectively, these data collected in vivo and in vitro support that the STAT3 pathway played a pivotal role in Tregs–microglia interactions.

### 3.6 STAT3 inhibitor suppressed microglia‐mediated neuroinflammation and promoted neural function recovery after SCI in Treg‐depleted mice

**FIGURE 6:**
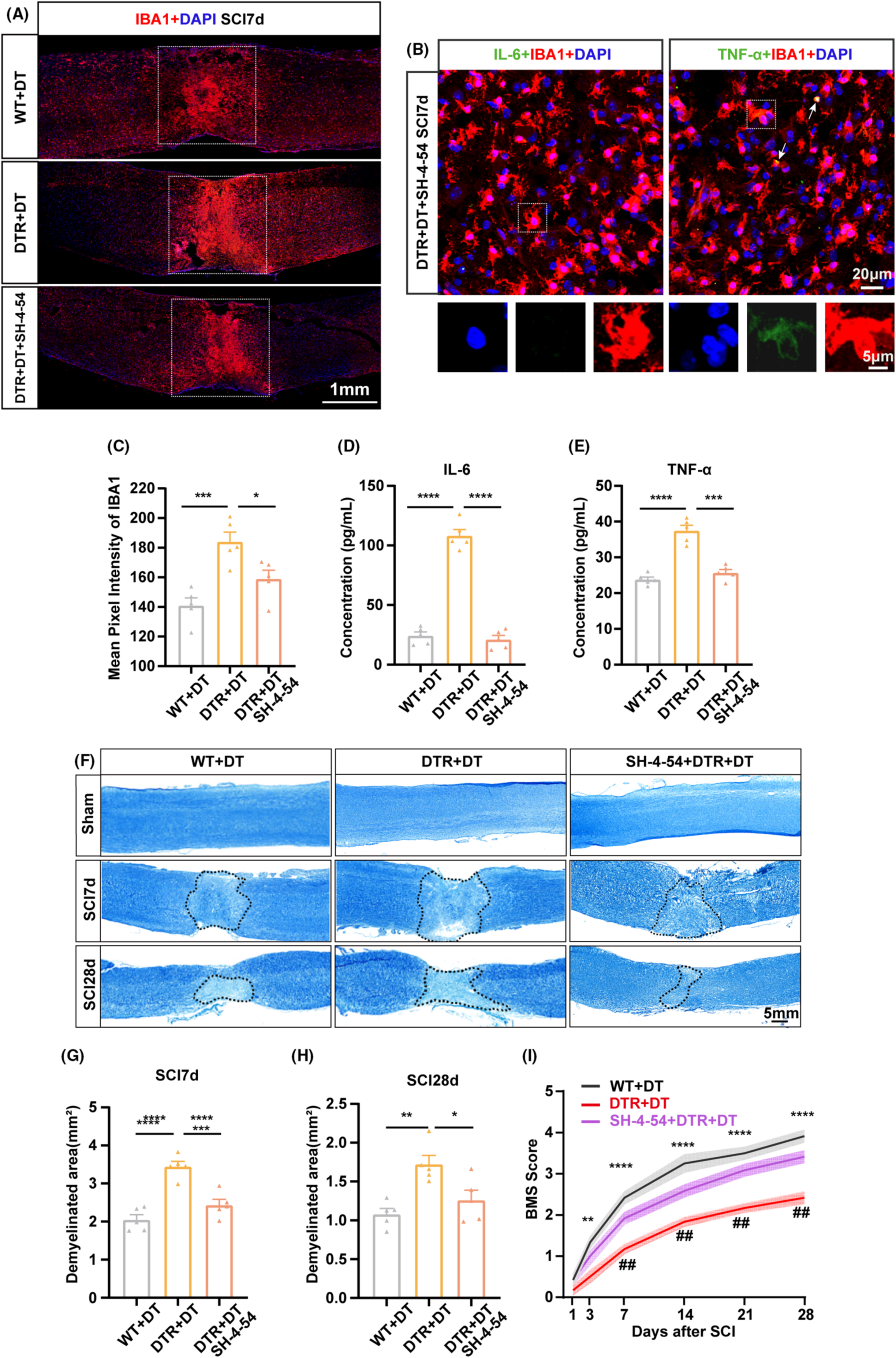
STAT3 inhibitor suppressed microglia‐mediated neuroinflammation and promoted neural function recovery in Treg‐depleted mice after SCI. (A) Representative images of spinal cord sections from DT‐treated WT (WT + DT) or *Foxp3‐DTR* mice, treated (DTR + DT + SH‐4‐54) or not (DTR + DT) with SH‐4‐54 STAT3 inhibitor, and stained for IBA1 (red) at day 7 post‐SCI. The white frames delineate region of interest (ROI), including the injury core and surrounding area. Scale bar: 1 mm. (B) Representative images of an injured spinal cord section from a DT‐treated *Foxp3‐DTR* mouse that received SH‐4‐54 STAT3 inhibitor, taken at day 7 post‐SCI (SCI7d). Double‐labeled cells for IL‐6 or TNF‐a (green) and IBA1 (red), are marked by white arrows. Scale bar: upper row, 20 μm; bottom row, 5 μm. (C) Summary of the percentage of IBA1^+^ area in ROI from mice described in (A). (D, E) Quantification by ELISA of IL‐6 (D) and TNF‐α (E) concentration in injured spinal cords from mice described in (A). (F) Representative images of LFB‐stained spinal cord coronal sections from sham‐operated mice, and mice treated as described in (A), which suffered SCI 7 (SCI7d) or 28 (SCI28d) days earlier. Scale bar: 5 mm. (G, H) Quantification of demyelinated area in SCI7d (G), and SCI28d (H) sections, as described in (F). (I) Total BMS scores at different time points following SCI in mice described in (A) (*n* = 10 for each group; **p < 0.01, ***p < 0.005, ****p < 0.001 for WT + DT vs. DTR + DT group; ^##^ *p* < 0.01, ^###^ *p* < 0.005, ^####^ *p* < 0.001 for DTR + DT + SH‐4‐54 vs. DTR + DT group). Data are represented as the mean ± SEM. For panel A–H, *n* = 5 for each group; *p < 0.05, **p < 0.01, ***p < 0.005, ****p < 0.001.

We treated *Foxp3‐DTR* mice with SH‐4‐54. Western blot analysis confirmed STAT3 inhibition by SH‐4‐54 treatment (Figure S3). Next, we evaluated the effect of STAT3 inhibition on microglia activation. As shown in Figure 6A, microglia density was significantly elevated in Treg‐depleted mice, whereas SH‐4‐54 treatment reduced the microglial population (*p* < 0.05; Figure 6C). Moreover, STAT3 inhibition resulted in downregulated IL‐6 and TNF‐α expression both in microglia (Figure 6B) and injured tissue (*p* < 0.005; Figure 6D,E) in Treg‐depleted mice.

Following SCI, a complex cascade of oxidative stress and inflammatory responses is initiated, leading to further demyelination, which is the major pathogenesis factor after SCI.^42^ To investigate whether Treg depletion promotes secondary tissue damage after SCI, we conducted LFB staining and observed that Treg depletion resulted in increased demyelination at both 7 and 28 days after SCI, while SH‐4‐54 treatment partly reversed this detrimental demyelination (*p* < 0.01; Figure 6F–H). Motor function recovery was evaluated by the BMS.^43^ In our study, all of the mice lost the motility of hind limbs and were rated 0 in BMS testing immediately after SCI, while the animals subject to sham surgery were rated 9 in BMS testing. One day after contusive injury, no significant locomotor recovery was observed. We noted that 72.2% of the injured mice did not have any motor function recovery (BMS score 0), whereas 27.8% of the animals subject to SCI exhibited slight ankle movement (BMS score 1) on day one after injury. Aggravated neural function deficits were then obvious after Treg depletion from day 3 after SCI onward (**p < 0.01; Figure 6I). STAT3 inhibition significantly ameliorated locomotor recovery from day seven after SCI, as visualized by markedly elevated BMS scores in SH‐4‐54‐treated Treg‐depleted mice compared to untreated counterparts (##*p* < 0.01; Figure 6I). These results showed that STAT3 inhibition suppressed microglial inflammatory response and partly reversed the neural deficits caused by Treg depletion.

## 4 DISCUSSION

Following SCI, the inflammatory microenvironment becomes dominant and damages neural regeneration and functional recovery.^4^, ^44^ New repair strategies for SCI have highlighted the importance of creating a microenvironment that prevents neuroinflammation and facilitates axonal outgrowth. Tregs are involved in maintaining immune homeostasis and suppressing neuroinflammation in the pathophysiological conditions affecting the CNS.^45^ Previous studies have reported that increasing Treg population promoted locomotor recovery after SCI.^24^, ^25^, ^26^ However, whether and how Tregs regulate the injured microenvironment after SCI has remained largely unknown.

Our data showed that the number of Tregs increased at least until day 28 after SCI, with a peak of infiltrate in peri‐injury areas on day 7. Previous studies have reported that Treg accumulation in ischemic brain is essential for white matter integrity after stroke.^21^ Our study showed that infiltrated Treg clustered around microglia and regulated microglia activation after SCI. In the context of SCI where Tregs were depleted, microglia acquired an amoeboid phenotype characterized by enlarged and densely stained soma with few short processes. Furthermore, Treg‐depletion resulted in increased proliferation capability by microglia and a switch toward a more proinflammatory phenotype on day 7 after SCI. These findings are consistent with previous reports in ALS and stroke, which indicated that Tregs regulate microglia activation and skews their differentiation toward an anti‐inflammatory and reparative phenotype.^21^, ^46^, ^47^, ^48^ A study in vitro demonstrated that Tregs suppress microglial synthesis and release of reactive oxygen species induced by misfolded α‐Synuclein, a protein associated with Parkinson's disease.^49^ Altogether, these results support that Tregs inhibit microglia proinflammatory activation after SCI.

Microglia play a central role in neuroinflammation through the release of multiple cytokines and chemokines.^50^ Importantly, our findings indicated that Tregs may improve the injured spinal microenvironment through regulating microglia activation. Several inflammation‐related genes, including *ApoD*, *Elane*, *Ctsg*, and *Cd200r3*, were upregulated in microglia in Treg‐depleted mice. These differentially expressed genes have been reported to promote the release of multiple cytokines, including CCL2, GM‐CSF, IL‐1β, IL‐2, IL‐6, IL‐33, MMP‐9, and TNF‐α, and therefore, to perpetrate an inflammatory microenvironment.^39^, ^51^, ^52^, ^53^, ^54^, ^55^, ^56^ Luminex analysis showed a robust increase in some of these proinflammatory cytokines (GM‐CSF, TNF‐α, IL‐1β, IL‐6, CCL2, and MMP‐9) and a reciprocal decrease in anti‐inflammatory cytokines (IL‐2 and IL‐33) in the injured environment after Treg depletion. These findings implied that Treg depletion promoted the development of a microglia‐mediated inflammatory environment after SCI. Similarly, recent studies have reported that Treg depletion almost completely abrogated the protective mechanisms against injury, through enabling TNFα, IL‐1β, IL‐6, and IL‐17 levels to increase rapidly.^57^ Conversely, the expansion of brain‐resident Tregs protects against pathological neuroinflammation.^58^ Collectively, these results suggest that Tregs inhibit microglia inflammatory response after SCI.

We next investigated the potential mechanism of microglia immunoregulation by Tregs. Transcriptome analysis showed that *ApoD* was a major gene significantly upregulated in microglia after Treg depletion. *ApoD* is mainly expressed in the central nervous system, and transcriptionally modifies the responses to oxidative stress and demyelination.^59^ Recent studies have shown that *ApoD* enhances inflammation by promoting the production of TNF‐α and IL‐6.^39^, ^60^ Interestingly, Tregs depletion led to TNF‐α and IL‐6 upregulation in injured spinal cord. Moreover, immunofluorescence histology showed that TNF‐α and IL‐6 were mostly colocalized with microglia, indicating that Tregs may mainly regulated the expression of TNF‐α and IL‐6 by microglia.

TNF‐α and IL‐6 are involved in canonical STAT3 activation signaling, which eventually impairs function recovery in numerous neuroinflammatory diseases.^29^, ^40^ In our study, we first noted a significant upregulation of phosphorylated STAT3 and *Socs3* mRNA in Treg‐depleted mice, suggesting an alteration of STAT3 activation status after Treg depletion. In vitro, OGD/R‐induced STAT3 activation was abolished in microglia cocultured with activated Tregs and by treatment with the STAT3 inhibitor SH‐4‐54. Our results strongly support that Tregs regulated microglial inflammatory response by suppressing the STAT3 pathway. This hypothesis was further supported in vivo, as STAT3 inhibition decreased microglia immunoreactivity and demyelination, and improved motor function in Treg‐depleted mice.

How Tregs modulate STAT3 signaling in microglia after SCI remains to be elucidated. It has been reported that Tregs maintain anti‐inflammatory microglial phenotypes through the release of IL‐4, IL‐10, IL‐35, and TGF‐β.^23^, ^46^, ^49^, ^61^ IL‐10 derived from Tregs has been previously reported to suppress the STAT3 pathway and ameliorate neuroinflammation in neuromyelitis optica spectrum disorder.^62^ IL‐35 enhanced M2 macrophage polarization through the STAT3 pathway.^63^ A recent study demonstrated that brain‐infiltrating Tregs express a higher level of osteopontin (OPN).^21^ OPN could modulate the STAT3 signaling pathway and promote macrophage M2 polarization.^64^, ^65^ To discover how Tregs modulate STAT3 signaling in microglia after SCI, further studies are needed including transcriptomic analyses and sub‐clustering analysis of infiltrated Tregs.

Indeed, astrocytes also play diverse roles in neuroinflammation after SCI.^66^ In our study, there was no significant change in astrocytes activation in Treg‐depleted mice, while other studies have elucidated that Tregs are involved in inhibiting astrocyte activation during sepsis‐associated encephalopathy and stroke.^27^, ^67^, ^68^ This discrepancy might be ascribed to different disease model and the time point we chose to observe, since astroglia rapidly increased and peaked around day one post‐SCI, followed by a decrease and steady level maintenance until day 42.^5^ Treg–astrocytes interaction after SCI in acute phase needs to be further investigated.

In summary, we showed that Treg–microglia crosstalk, likely involving STAT3, plays a pivotal role in neuroinflammation resolution after SCI. STAT3 inhibition could attenuate functional deficits in Treg‐depleted mice. These results support a critical contribution of endogenous Tregs to reducing the inflammatory response and thus improving functional recovery after SCI. Our findings may provide novel insights into more specific Treg‐associated mechanisms underlying neuronal protection and open possibilities of therapeutic development for SCI.

## AUTHOR CONTRIBUTIONS

RL, YL, ZYW, PC, and YX carried out the experiments. RL, YL, ZYY, and ZYW participated in the data acquisition and analysis. RL, ZYY, and MHW wrote the manuscript and revised the manuscript. XL designed the experiments and reviewed the paper. All authors read and approved the final manuscript.

## FUNDING INFORMATION

This study was supported by the National Nature Science Foundation of China (82171385 to X. Luo), the Key Research and Development Program of Hubei Province (2020BCA070 to X. Luo), the Application Foundation Frontier Special Project of Wuhan Science and Technology Bureau (2020020601012226 to X. Luo), and Ministry of Science and Technology China Brain Initiative Grant (2022ZD0204704 to W. Wang).

## CONFLICT OF INTEREST STATEMENT

The authors declare no competing interest.

## Supporting information


Appendix S1.

Figure S1.

Figure S2.

Figure S3.

Table S1.

Table S2.

## Data Availability

The data that support the findings of this study are available from the corresponding author upon reasonable request.
