# Supplementary material for: Regulatory T cells promote functional recovery after spinal cord injury by alleviating microglia inflammation via STAT3 inhibition
Source: CNS Neurosci Ther. 2023 Mar 13;29(8):2129–44. doi: 10.1111/cns.14161 (PMC10352886; doi:10.1111/cns.14161)
Supplement: Supplementary file 1 — Appendix S1. [file CNS-29-2129-s004.docx]

**Three-dimensional reconstruction and measurement of the co-localization coefficient**

The image-processing software Imaris (Bitplane) was used to reconstruct three-dimensional images of cells as described (1). Z-stack confocal images were imported into Imaris, and the surface module was used to generate 3D structures of each color channel. Region of interest was selected, and the absolute intensity of each source channel was used for reconstruction. Smoothing was set at 0.24 μm for all channels and images. A threshold was set to differentiate the target signal from background, and the same threshold value was used for all of the groups. Nonspecific signals were removed, and the 3D-rendered images were constructed. The mask module was used to calculate the co-localization coefficient. (2) For each channel, a masked surface was then built. The voxels outside the surface were set at 0, while the voxels inside the surface were set at 100. The co-localization coefficients of two masked surfaces were defined by the build-coloc-channel modules of the software.

All images were captured using the same camera and microscope settings and were processed with the same adjustments and parameters.

**Sholl analysis**

Morphological changes in microglia were analyzed according to the Sholl method. Namely, total branch length and number of branching points were measured with the Sholl analysis plugin for ImageJ. To this end, microglial cells were traced at a 600× magnification, and Sholl created a series of concentric circles around the cell bodies. The starting radius was 3.00 µm, and the radius interval between the circles was 2 µm. The number of branch intersections with the concentric circles was counted.

**Basso Mouse Scale**

Locomotion recovery following spinal cord injury was assessed with the BMS by measuring hind limb joint activities, trunk position and stability, front and rear limbs coordination, paw position, toe clearance, and tail position. (3) Successful contusion model resulted in complete motor function loss of the hind limbs 2 h after injury (BMS score 0, no ankle movement), whereas successful sham surgery models had no effect on hind limb mobility (BMS score 9, frequent or consistent plantar stepping, mostly coordinated, paws parallel at initial contact and lift off, normal trunk stability, and tail always up).

The scores were recorded before surgery and at 1, 3, 7, 14, 21, and 28 days after SCI. The mice were placed in an open field, and the activities were observed and recorded by two investigators blinded to the treatment group.

**Table 1 Catalog Number of Antibodies**

| **Antibody** | **SOURCE** | **Catalog Number** |
| --- | --- | --- |
| CD16/32 (1:200) | BD | 553140 |
| CD4 (1:200) | BD | 553051 |
| CD25 (1:200) | BD | 553075 |
| CD45 (1:200) | BD | 550994 |
| CD11b (1:200) | BD | 553312 |
| IBA1 (1:200, host: rabbit) | Wako | 019-19741 |
| IBA1 (1:200, host: goat) | Wako | 011-27991 |
| GFAP (1:200) | Abcam | 302644 |
| NeuN (1:200) | CST | 36662 |
| KI67 (1:200) | Abcam | 156956 |
| CD16 (1:200) | Boster | A01408 |
| CD206 (1:200) | R&D | AF2535 |
| IL-6 (1:200) | Santa Cruz | 32296 |
| TNF-α (1:200) | Santa Cruz | 12744 |
| STAT3 (1:1000) | CST | 12640S |
| Phosphor-STAT3 (1:1000) | CST | 9145S |
| β-Actin | Servicebo | 15001 |
| GAPDH | Servicebo | 15002 |
| CD3 | Thermo Fisher | 14-0031-82 |

**References**

1. Shi L, Sun Z, Su W, Xu F, Xie D, Zhang Q, et al. Treg cell-derived osteopontin promotes microglia-mediated white matter repair after ischemic stroke. Immunity. 2021;54(7):1527-42 e8.

2. Ducza L, Szucs P, Hegedus K, Bakk E, Gajtko A, Weber I, et al. NLRP2 Is Overexpressed in Spinal Astrocytes at the Peak of Mechanical Pain Sensitivity during Complete Freund Adjuvant-Induced Persistent Pain. Int J Mol Sci. 2021;22(21).

3. Basso DM, Fisher LC, Anderson AJ, Jakeman LB, McTigue DM, Popovich PG. Basso Mouse Scale for locomotion detects differences in recovery after spinal cord injury in five common mouse strains. J Neurotrauma. 2006;23(5):635-59.
