# Supplementary figures and images for: Regulatory T cells promote functional recovery after spinal cord injury by alleviating microglia inflammation via STAT3 inhibition
Source: CNS Neurosci Ther. 2023 Mar 13;29(8):2129–44. doi: 10.1111/cns.14161 (PMC10352886; doi:10.1111/cns.14161)

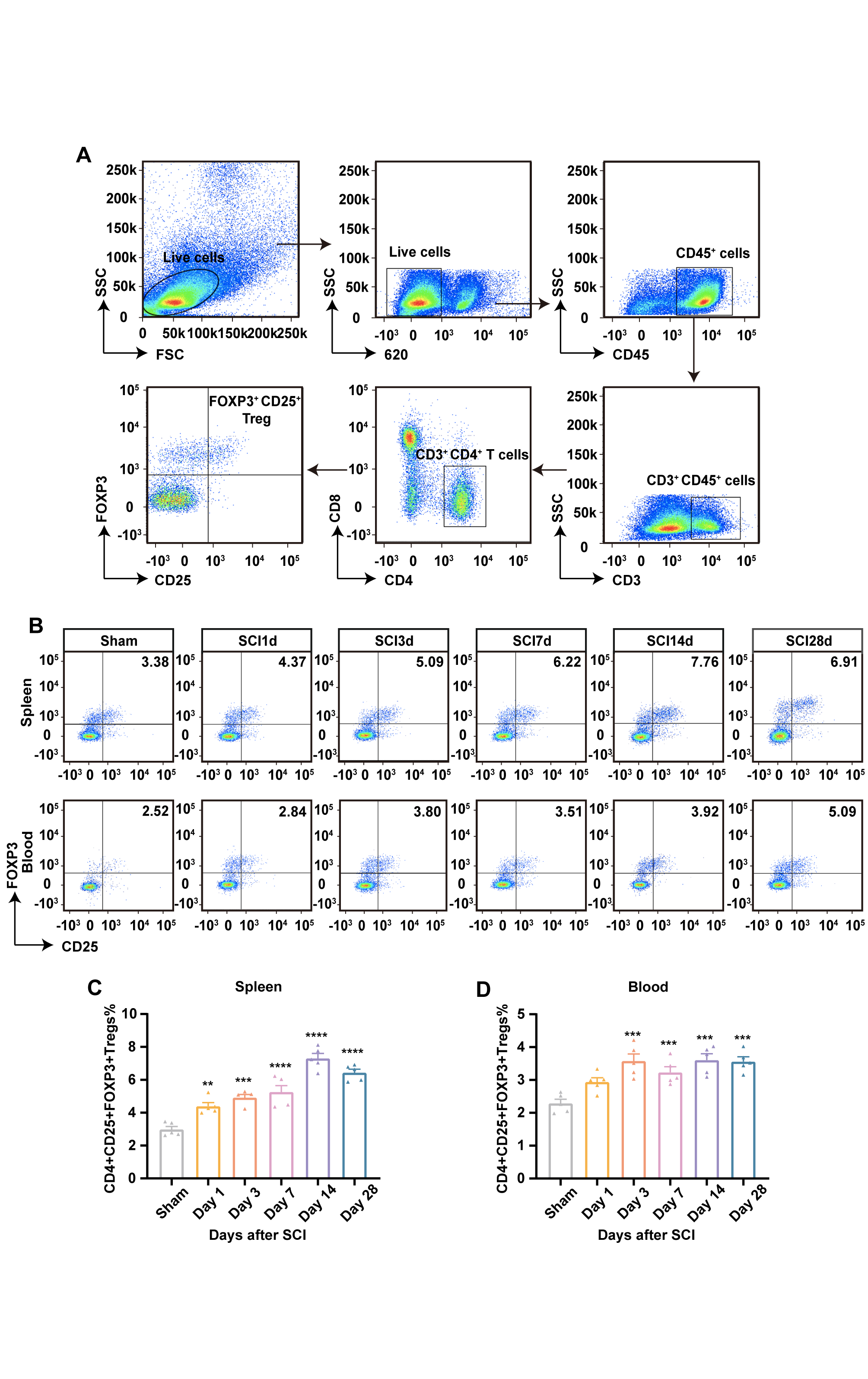

Supplement: Supplementary file 2 — Figure S1. [file CNS-29-2129-s002.tif]

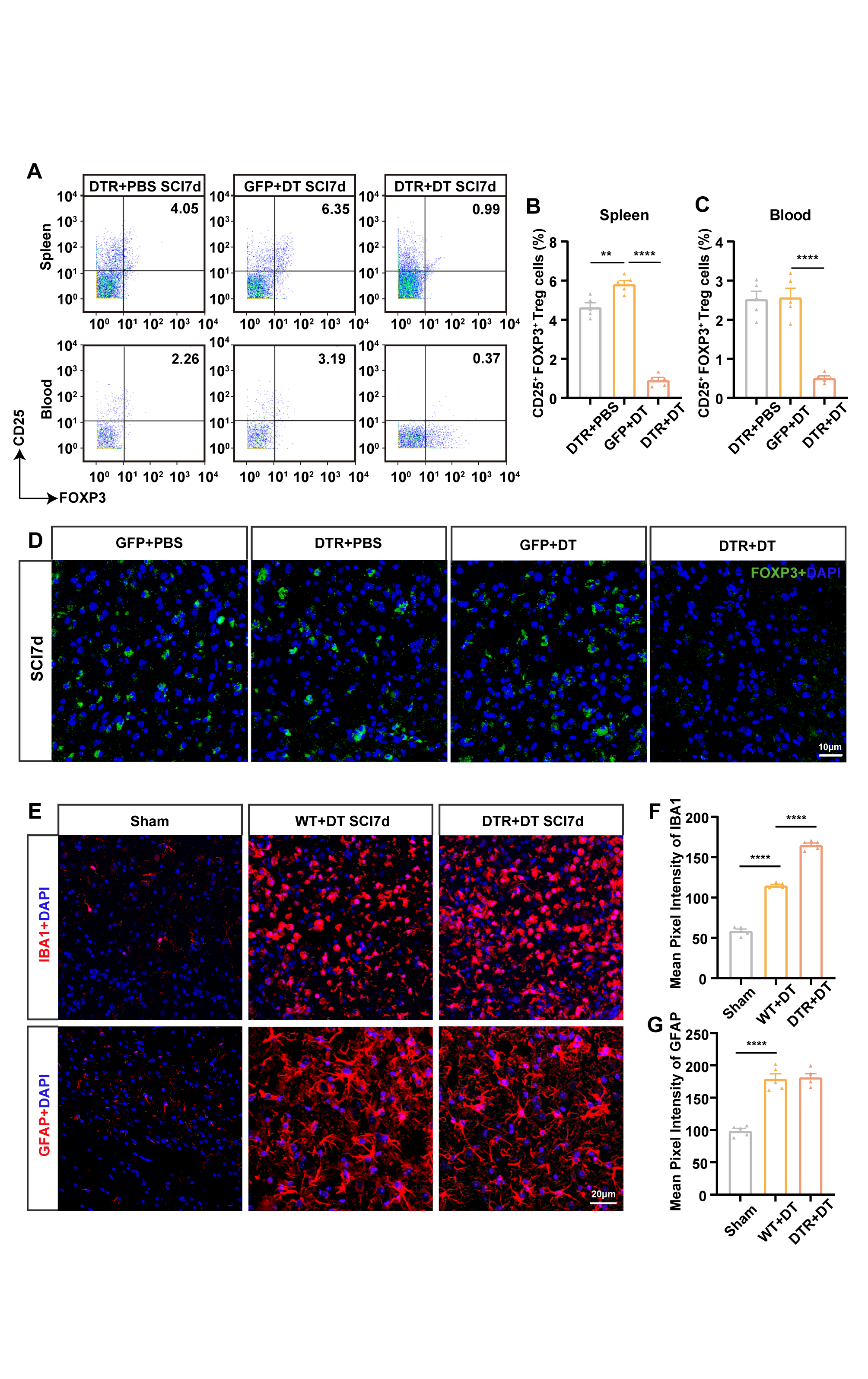

Supplement: Supplementary file 3 — Figure S2. [file CNS-29-2129-s006.tif]

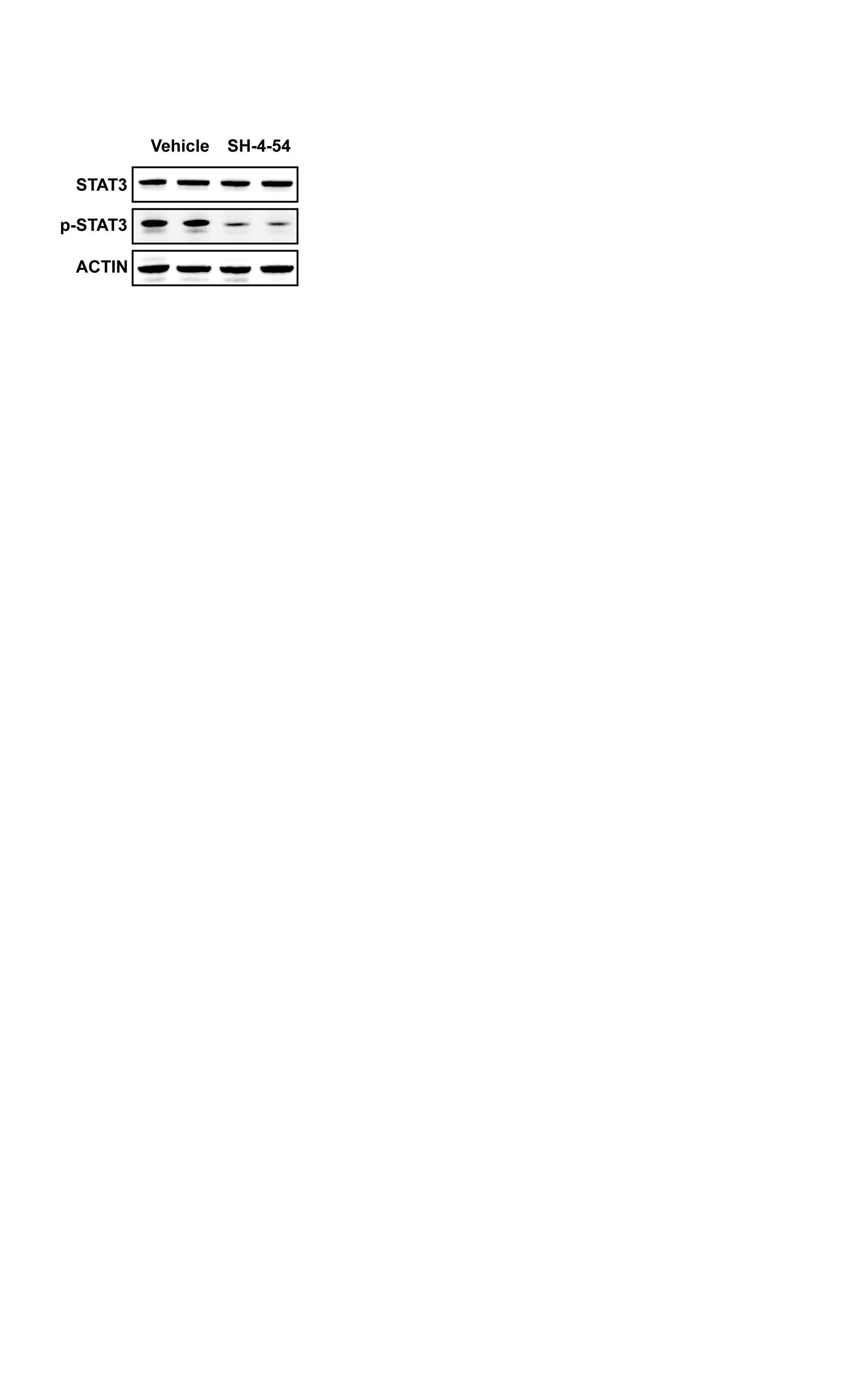

Supplement: Supplementary file 4 — Figure S3. [file CNS-29-2129-s005.tif]
