# Supplementary material for: Regulatory T cells promote functional recovery after spinal cord injury by alleviating microglia inflammation via STAT3 inhibition
Source: CNS Neurosci Ther. 2023 Mar 13;29(8):2129–44. doi: 10.1111/cns.14161 (PMC10352886; doi:10.1111/cns.14161)
Supplement: Supplementary file 5 — Table S1. [file CNS-29-2129-s003.docx]

**Table S1 Catalog Number of Antibodies**

| **Antibody** | **SOURCE** | **Catalog Number** |
| --- | --- | --- |
| CD16/32 (1:200) | BD | 553140 |
| CD4 (1:200) | BD | 553051 |
| CD25 (1:200) | BD | 553075 |
| CD45 (1:200) | BD | 550994 |
| CD11b (1:200) | BD | 553312 |
| IBA1 (1:200, host: rabbit) | Wako | 019-19741 |
| IBA1 (1:200, host: goat) | Wako | 011-27991 |
| GFAP (1:200) | Abcam | 302644 |
| NeuN (1:200) | CST | 36662 |
| KI67 (1:200) | Abcam | 156956 |
| CD16 (1:200) | Boster | A01408 |
| CD206 (1:200) | R&D | AF2535 |
| IL-6 (1:200) | Santa Cruz | 32296 |
| TNF-α (1:200) | Santa Cruz | 12744 |
| STAT3 (1:1000) | CST | 12640S |
| Phosphor-STAT3 (1:1000) | CST | 9145S |
| β-actin | Servicebo | 15001 |
| GAPDH | Servicebo | 15002 |
| CD3 | Thermofisher | 14-0031-82 |
