# Supplementary material for: Regulatory T cells promote functional recovery after spinal cord injury by alleviating microglia inflammation via STAT3 inhibition
Source: CNS Neurosci Ther. 2023 Mar 13;29(8):2129–44. doi: 10.1111/cns.14161 (PMC10352886; doi:10.1111/cns.14161)
Supplement: Supplementary file 6 — Table S2. [file CNS-29-2129-s001.docx]

**Table S2. Primer sequences**

| Gene name | Forward primer |
| --- | --- |
| *ApoD* | 5‘-GAAACTGCATTCAAGCCAACTA-3’ |
|  | 3‘-TACTTGGTTCATGGTTCCATCA-5’ |
| *Elane* | 5‘-CTGTGTGAACGGCCTAAATTTC-3’ |
|  | 3‘-CAGAGAAGGTCTGTCGAGTG -5’ |
| *Cd200r3* | 5‘-GTCCATCAAAAGAAGCAACACT-3’ |
|  | 3‘-TTTACTGATCTGCTGCAACTCT-5’ |
| *Ctsg* | 5‘-ACAACATCCAAATGCGAGAAAG-3’ |
|  | 3‘-CATGATGTCATTCCGGATGTTC-5’ |
| *Ctsk* | 5‘-GCTTGGCATCTTTCCAGTTTTA-3’ |
|  | 3‘-CAACACTGCATGGTTCACATTA-5’ |
| *Ambp* | 5‘-TTCATAAAGCTCTGGGCATTTG-3’ |
|  | 3‘-CTCAGAGTAGAATTTGTTGCCG-5’ |
| *Tnfa* | 5‘-ATGTCTCAGCCTCTTCTCATTC-3’ |
|  | 3‘-GCTTGTCACTCGAATTTTGAGA-5’ |
| *Nox2* | 5‘-GACAGGAACCTCACTTTCCATA-3’ |
|  | 3‘-TGAAGAGATGTGCAATTGTGTG-5’ |
| *Il6* | 5‘-CTCCCAACAGACCTGTCTATAC-3’ |
|  | 3‘-CCATTGCACAACTCTTTTCTCA-5’ |
| *Ccl3* | 5‘-TTGCTGTTCTTCTCTGTACCAT-3’ |
|  | 3‘-AATAGTCAACGATGAATTGGCG-5’ |
| *Socs3* | 5‘-GACCAAGAACCTACGCATCCAGTG-3’ |
|  | 3‘-GCACCAGCTTGAGTACACAGTCG-5’ |
